# Diagnostic wrist arthroscopy: findings in patients suspected of TFCC lesions

**DOI:** 10.1007/s00402-025-06002-8

**Published:** 2025-08-06

**Authors:** Lyse van Wijk, Sandesh Kasie, Claire Koeyvoets, Sebastiaan Souer, Hand-Wrist Study Group, Steven Hovius, Brigitte van der Heijden

**Affiliations:** 1 Department of Plastic, Reconstructive and Hand Surgery, Radboud University Nijmegen Medical Centre, Nijmegen, Netherlands; 2 Hand and Wrist Center, Xpert Clinics, Amsterdam, Netherlands; 3 Department of Plastic, Reconstructive and Hand Surgery, Jeroen Bosch Ziekenhuis, ‘s-Hertogenbosch, Netherlands

## Abstract

**Introduction:**

Ulnar-sided wrist pain remains a diagnostic challenge due to its complex anatomy and broad differential diagnosis. This study evaluated the diagnostic role and therapeutic consequences of wrist arthroscopy in patients with ulnar-sided wrist pain and suspected triangular fibrocartilage complex (TFCC) lesions.

**Materials and methods:**

A retrospective cohort study was conducted on patients undergoing diagnostic wrist arthroscopy for suspected TFCC lesions based on clinical and imaging assessments between 2012 and 2021. Data on the indication, arthroscopic findings, treatment, and complications were collected.

**Results:**

500 patients were included with a median age of 38 years (IQR 28.0–49.0), of whom 65.0% were women. The median symptom duration was 8 months (IQR 5.0-16.5), with 61.4% reporting wrist trauma. TFCC lesions were confirmed in 73.6% of cases, most commonly Palmer type 1B and 2C. Additional findings, such as scapholunate or lunotriquetral injuries and cartilage damage, were identified in 31.6% of patients, while 16.0% showed no arthroscopic abnormalities. Based on arthroscopic findings, 46.4% underwent immediate arthroscopic intervention, including TFCC debridement (174, 34.8%), synovectomy (170, 34.0%), or ganglion removal (4, 0.8%). 51.0% proceeded to open surgery within a year, most often TFCC repair or ulnar shortening. Complications occurred in 7.4% of patients, mostly mild.

**Conclusions:**

These results highlight wrist arthroscopy as a decisive tool in the diagnostic workup of patients with suspected TFCC lesions, providing valuable information to confirm, classify, or exclude pathology, and to identify concomitant wrist abnormalities. Both positive and negative arthroscopic findings are important for treatment decisions, providing essential guidance for surgical and non-surgical management of ulnar-sided wrist pain.

## Introduction

Ulnar-sided wrist pain remains a diagnostic challenge, often referred to as the “headache of the wrist” due to its complex anatomy and broad differential diagnosis [1]. Among the various causes, triangular fibrocartilage complex (TFCC) injuries are frequently encountered in clinical practice. However, accurately identifying and determining the clinical relevance of TFCC lesions remains difficult [2]. Clinical examination tests have limited sensitivity and specificity in detecting TFCC pathology [3–5]. Imaging modalities like magnetic resonance imaging (MRI) and MR arthrography (MRA) have moderate diagnostic accuracy, with reported sensitivities and specificities of 0.76–0.85 in studies that use arthroscopy as the reference standard [6–9].

Despite advancements in non-invasive diagnostics, wrist arthroscopy continues to be considered the reference for direct visualization and dynamic assessment of intra-articular structures, including the TFCC. Arthroscopy allows for probing and evaluation of ligament integrity, which is not possible with static imaging techniques. However, it is important to acknowledge the limitations of arthroscopy, including its invasive nature, relatively high costs, risk of complications and a notable degree of interobserver variability [10–13].

Given these complexities, there is scientific and clinical value in analyzing the arthroscopic findings and their impact on subsequent treatment decisions in a cohort of patients presenting with ulnar-sided wrist pain. This article aims to contribute to the understanding of the diagnostic role and resulting treatments of wrist arthroscopy in this difficult patient population. It describes the findings during wrist arthroscopy and following treatment in patients with ulnar sided wrist pain, suspected for TFCC pathology.

## Methods

We conducted a retrospective cohort study of patients undergoing diagnostic wrist arthroscopy between 2012 and 2021 at < blinded > in < blinded>. < Blinded > comprises 28 clinics providing specialized hand and wrist care. Upon undergoing treatment and providing informed consent, patients have their measurements automatically recorded and distributed via a secure online system [14]. The exact setting of this cohort has previously been described [15]. We reviewed medical records for information about the indication of arthroscopy, wrist trauma in history, the arthroscopic procedure and findings, and follow-up treatment.

We included all patients aged 18 years or older who underwent diagnostic wrist arthroscopy because of ulnar-sided wrist pain suspected for a TFCC lesion. Clinical suspicion of a TFCC lesion was based on medical history, physical examination, and imaging, as determined by the treating clinician. Due to the observational study design, no standardized diagnostic protocol was applied. Patients without an available arthroscopy report were excluded.

The ethics committee of our institution approved the study protocol, and all patients provided informed consent. The study adhered to the Strengthening The Reporting of OBservational studies in Epidemiology (STROBE) statement [16].

For the arthroscopic findings, we collected data from standard procedure reports. For each patient, we assessed the findings of the TFCC, scapholunate ligament (SLL), lunotriquetral ligament (LTL), radioscapholunate ligament (RSL), ganglion, synovitis, and cartilage damage. Cartilage damage included any damage to the scaphoid (fossa), lunate (fossa), capitate, triquetrum, and hamate. In this standard procedure report, TFCC lesions were reported according to the Palmer classification [17]. This classification was used solely to describe the anatomical location of the TFCC lesion, not to infer its etiology. It served as a topographical reference and does not distinguish between traumatic and degenerative origins. SLL and LTL lesions were assessed according to the Geissler classification [18], and cartilage damage was noted but not graded. We are aware of the newer arthroscopic classifications for ligament lesions [19, 20]; however, these classifications were not available at the time slot of this study and therefore not used in our clinics then. We also gathered data on type of anaesthesia, tourniquets, and portals used.

All wrist arthroscopies in our study were performed by hand surgeons at levels III-IV, as classified by the Tang and Giddins system [21]. Patients were positioned supine, with the wrist placed under traction. The 3–4, 4–5, or 6R portals were used for the radiocarpal joint, and the midcarpal radial (MCR) and midcarpal ulnar (MCU) portals for the midcarpal joint. A 1.9 mm arthroscope from Arthrex GmbH was used.

To gain insight into the treatment decisions after arthroscopy, we report the treatments of all patients within one year after diagnostic wrist arthroscopy, including non-surgical treatment, arthroscopic treatment, and open surgery.

Complications were retrospectively extracted from the electronic patient records. Complications were defined as any deviations from the normal arthroscopic procedure or recovery within three months.

Descriptive statistics were utilized for outcome analysis. Continuous data distribution was assessed with histograms and QQ plots. Normally distributed data were reported as means with standard deviations (SD), while skewed data were presented as medians with interquartile ranges (IQR). Categorical variables were summarized using numbers and percentages. Statistical analyses were conducted using R (version 4.3.1).

## Results

**Fig. 1:**
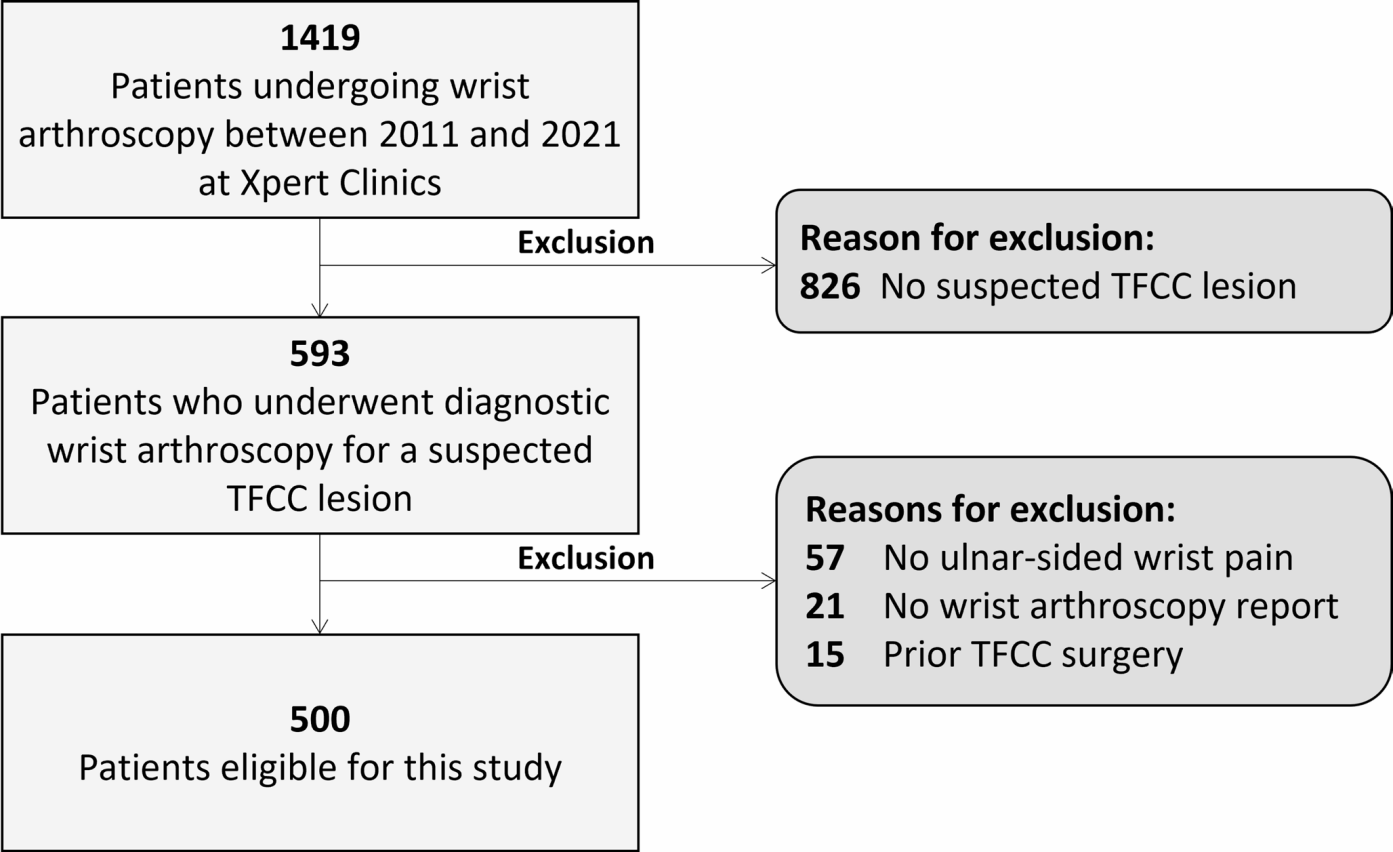
Flowchart of patient selection

A total of 585 patients underwent diagnostic arthroscopy for suspected TFCC lesion. After applying the exclusion criteria, 500 patients were eligible to be included in this study (Fig. 1).

### Patient characteristics

Table 1 summarizes the demographic and clinical characteristics of the patient cohort. The median age of the patients was 38 years (interquartile range [IQR] 28.0–49.0), with a majority being female (65.0%). The median duration of complaints was 8 months (IQR 5.0-16.5), and 307 (61.4%) patients recalled having had a traumatic injury to the wrist.

**Table 1:** Baseline characteristics of patients undergoing wrist arthroscopy for ulnar-sided wrist pain suspected of a TFCC lesion

Variables	*N* = 500	Missing (%)
Age, median [IQR]	38 [28.0, 49.0]	
Sex = Male, n (%)	175 (35.0)	
Duration of symptoms in months, median [IQR]	8 [5.0, 16.5]	
Dominant side affected = Yes, n (%)	277 (58.2)	4.8
Type of work, n (%)		
*None*	105 (21.0)	
*Light*	153 (30.6)	
*Medium*	165 (33.0)	
*Heavy*	77 (15.4)	
Previous non-surgical treatment = Yes, n (%)	402 (80.4)	
Previous surgical treatment = Yes, n (%)	46 (9.2)	
Trauma = Yes, n (%)	307 (61.4)	
Location of complaints = Ulnar-sided, n (%)	500 (100)	
MRI before arthroscopy = Yes, n (%)	133 (26.6)	
CT before arthroscopy = Yes, n (%)	10 (2.0)	
X-ray before arthroscopy = Yes, n (%)	385 (77.0)	
Arthroscopy portals, n (%)		
*Radiocarpal and midcarpal*	226 (45.2)	
*Radiocarpal*	274 (54.8)	
Anesthesia (%)		
*General anaesthesia*	27 (5.4)	
*Regional with sedation*	19 (3.8)	
*Regional without sedation*	454 (90.8)	
Tourniquet used = Yes, n (%)	427 (85.4)	

*IQR* interquartile range

Prior to arthroscopy, most patients had received non-surgical treatment (80.4%), while a smaller proportion had undergone hand surgery (9.2%) for non-related pathology, like carpal tunnel release. MRI was performed prior to arthroscopy in 133 patients (26.6%) with the results displayed in Table 2. Both radiocarpal and midcarpal portals were used in 45.2% of patients; in the remaining patients, only radiocarpal portals were used.

**Table 2:** Relationship between MRI and arthroscopic findings regarding the TFCC

		Arthroscopy	
		TFCC lesion	No TFCC lesion
MRI	TFCC lesion	61 (45.9)	14 (10.5)
	No TFCC lesion	30 (22.6)	28 (21.1)

Sensitivity: 0.67; Specificity: 0.67; Positive predictive value: 0.81; Negative predictive value: 0.48

### Arthroscopic findings

**Fig. 2:**
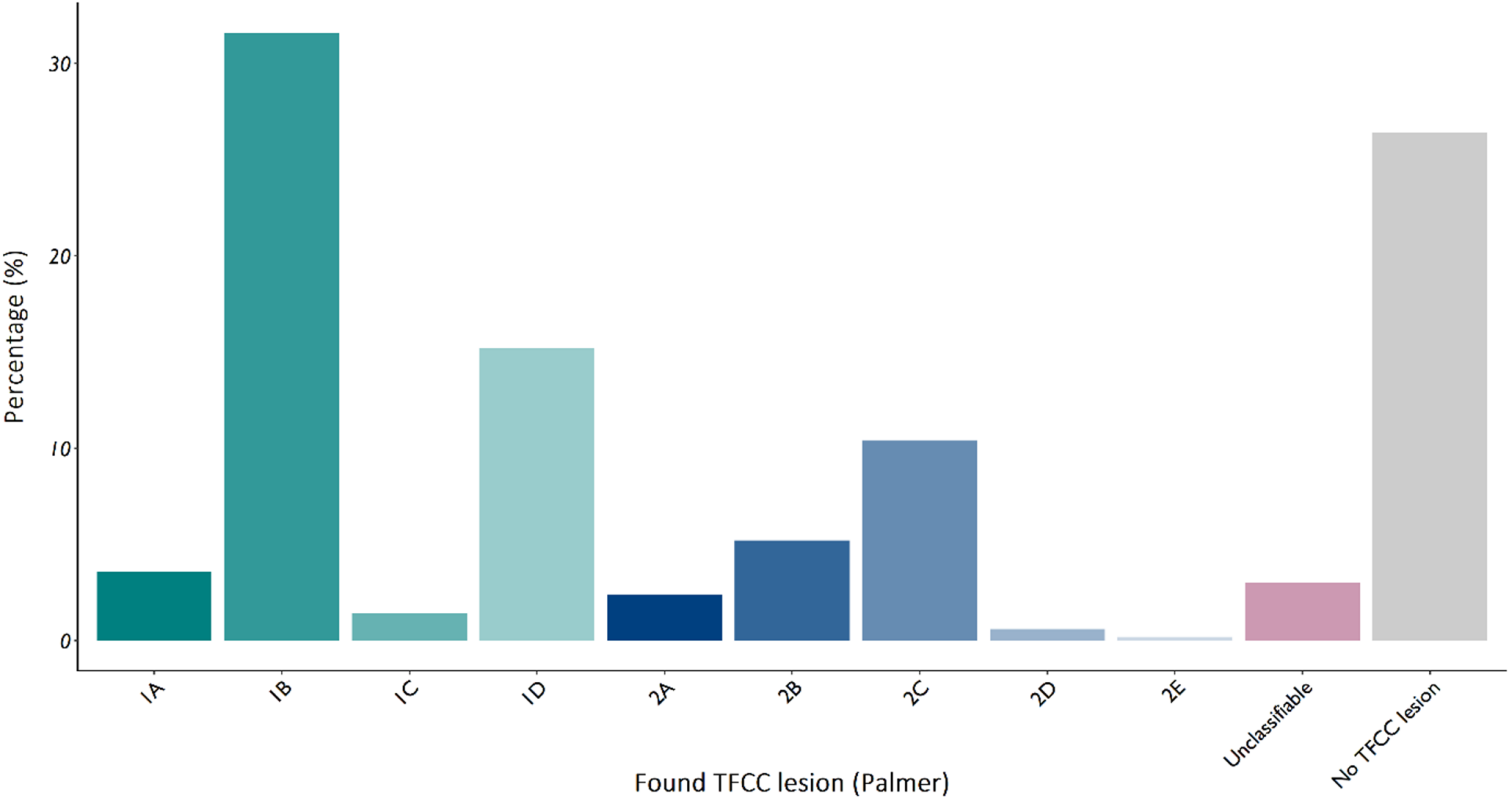
TFCC findings in patients with ulnar-sided wrist pain, suspected for a TFCC lesion, categorized in groups according to the Palmer grade (%)

Table 3 presents an overview of all arthroscopic findings. The suspicion of a TFCC lesion was confirmed by arthroscopy in 368 patients (73.6%), with Palmer 1B (31.6%), 1D (15.2%), and 2 C (10.4%) being the most classified lesions (Fig. 2). In 15 patients (3.0%), TFCC pathology was found not classifiable according to the Palmer classification. Following a recent classification [19], these lesions were R1 Deep lesions in 11 (2.2%) and W1 lesions in 4 (0.8%) patients.

Additional findings were identified in 31.6% of all patients, including SLL lesions (15.0%), LTL lesions (8.8%), and cartilage damage (21.2%) (Table 3). Cartilage damage was most prevalent around the lunate and lunate fossa. Additional findings were found across all types of TFCC lesions without a clear association.

Arthroscopy revealed no clear pathology in 80 patients (16.0%).

**Table 3:** Arthroscopic findings in patients with ulnar-sided wrist pain suspected for a TFCC lesion

Arthroscopic findings	*N* (%)
*Palmer*	
A	18 (3.6)
1B	158 (31.6)
1C	7 (1.4)
1D	76 (15.2)
2A	12 (2.4)
2B	26 (5.2)
2C	52 (10.4)
2D	3 (0.6)
2E	1 (0.2)
Other TFCC lesions*	15 (3.0)
*No TFCC lesion*	132 (26.4)
*SLL lesion*	75 (15.0)
Geissler I/II	34 (6.8)
Geissler III	29 (5.8)
Geissler IV	12 (2.4)
*LTL lesion*	44 (8.8)
Geissler I/II	25 (5.0)
Geissler III	12 (2.4)
Geissler IV	7 (1.4)
*RSL lesion*	20 (4.0)
*Ganglion*	5 (1.0)
*Synovitis*	215 (43.0)
*Cartilage damage*	106 (21.2)
Scaphoid	16 (3.2)
Scaphoid fossa	26 (5.2)
Lunate	65 (13.0)
Lunate fossa	32 (6.4)
Triquetrum	18 (3.6)
Capitate	6 (1.2)
Hamate	8 (1.6)
*No findings*	80 (16.0)

*Unclassifiable according to Palmer; including R1 (*n* = 11) and W1 (*n* = 4) lesions according to Herzberg et al.

*TFCC* Triangular Fibrocartilage Complex, *SLL* Scapholunate ligament, *LTL* Lunotriquetral ligament, *RSL* Radioscapholunate ligament.

### Treatment

Following the diagnostic part of the arthroscopy, 232 patients (46.4%) had one or more interventions during the same arthroscopy, including TFCC debridement (174, 34.8%), synovectomy (170, 34.0%), or ganglion removal (4, 0.8%).

Of all patients, 255 (51.0%) underwent open surgery either directly or within one year after arthroscopy (Table 4). Of all patients with a confirmed TFCC lesion, 57.1% underwent either a TFCC repair (42.1%), ulnar shortening (13.9%), or both (1.1%) (Table 5).

The 105 patients (21.0%) who did not undergo an arthroscopic procedure or open surgery, were treated with hand therapy and additional injections (16.2%) or splinting (20.0%).

**Table 4:** Surgical interventions within one year after diagnostic wrist arthroscopy

Treatment	*n* (%)
Total	255 (51.0)
TFCC repair	159 (31.8)
Ulnar shortening	56 (11.2)
ECU subsheath reconstruction	6 (1.2)
Extensor retinaculum capsulorrhaphy	3 (0.6)
LTL reconstruction	7 (1.4)
Three-ligament tenodesis	19 (3.8)
Dorsal capsular reinforcement	6 (1.2)
Proximal row carpectomy	1 (0.2)
Radioscapholunate arthrodesis	3 (0.6)
Pisiformectomy	8 (1.6)
Ulnar stylodectomy	2 (0.4)
Hamate removal	1 (0.2)
Wrist denervation	2 (0.4)
Wrist prothesis	1 (0.2)
Open exploration	3 (0.6)
Open synovectomy	5 (1.0)
Correction osteotomy radius	3 (0.6)

*TFCC* triangular fibrocartilage complex, *LTL* lunotriquetral ligament, *ECU* extensor carpi ulnaris.

**Table 5:** The number of arthroscopies followed by TFCC repair and/or ulnar shortening (US) within one year after confirmed TFCC lesion

Palmer	Number of TFCC lesions found	Number of patients who underwent surgery, *n* (%)	TFCC repair, *n* (%)	US, *n* (%)	TFCC repair and US, *n* (%)
1A	18	5 (27.8)	5 (27.8)	–	–
1B	158	111 (70.3)	110 (69.6)	–	1 (0.6)
1C	7	1 (14.3)	1 (14.3)	–	–
1D	76	32 (42.1)	27 (35.5)	5 (6.6)	–
2A	12	2 (16.7)	–	2 (16.7)	–
2B	26	14 (53.8)	–	13 (50.0)	1 (3.8)
2C	52	32 (61.5)	2 (3.8)	28 (53.8)	2 (3.8)
2D	3	3 (100.0)	1 (33.3)	2 (66.7)	–
2E	1	1 (100.0)	–	1 (100.0)	–
Other TFCC lesion	12	6 (50.0)	6 (50.0)	–	–
Total	368	210 (57.1)	155 (42.1)	51 (13.9)	4 (1.1)

### Complications

Among all patients, 7.4% experienced a complication during or following wrist arthroscopy (Table 6). In 11 patients, there was poor view during the procedure, requiring conversion to open exploration in three patients. Other frequently reported complications included hypoesthesia on the dorsal side of the wrist or fingers, as well as prolonged wrist stiffness. Additionally, one patient developed a pneumothorax following a brachial plexus block.

**Table 6:** Complications within three months after wrist arthroscopy or until follow-up surgery

Complications	*N* (%)
No view during arthroscopy	11 (2.2)
*No clear visualization due to synovitis*	*7 (1.4)*
*No access to the midcarpal portal possible*	*3 (0.6)*
*No access to the 6R portal possible*	*1 (0.2)*
Hypoesthesia	7 (1.4)
*Dorsal skin of the wrist*	*2 (0.4)*
*Digits 2 and 4*	*1 (0.2)*
*Digits 3 and 4*	*1 (0.2)*
*Digits 4 and 5*	*1 (0.2)*
*Digit 5*	*2 (0.4)*
Prolonged stiffness	6 (1.2)
Sensitivity loss	3 (0.6)
*Median nerve*	*2 (0.4)*
*Dorsal branch of radial and ulnar nerve*	*1 (0.2)*
Extra pain medication for high post-operative pain	2 (0.4)
Extensor pollicis longus inflammation	1 (0.2)
Fever after arthroscopy	1 (0.2)
Phlebitis due to intravenous line	1 (0.2)
Pneumothorax after brachial plexus block	1 (0.2)
Prolonged swelling	1 (0.2)
Reduced strength	1 (0.2)
Scar hypersensitivity	1 (0.2)
Ulnar neuropathy	1 (0.2)
Total	37 (7.4)

## Discussion

This study evaluated the diagnostic role of arthroscopy in patients with a suspected TFCC lesion. It demonstrates that wrist arthroscopy confirmed TFCC lesions in 73.6% of patients with clinical suspicion, while 26.4% had no TFCC lesion, 31.6% additional findings and 16.0% not any clear pathology, highlighting the limited specificity of clinical assessment and imaging, consistent with previous reports [3–9]. Importantly, negative arthroscopy findings do not equate to a negative outcome and significantly influences treatment by preventing unnecessary surgery and guiding non-surgical management. Moreover, in the majority of cases, the final treatment plan aligned with the arthroscopic findings, reinforces the role of arthroscopy as a decisive diagnostic tool.

Our findings align with prior research: Boer et al. reported TFCC lesions in 66.6% and Spies et al. in 61.3%, the latter reflecting broader inclusion criteria [22, 23]. Palmer type 1B was the most common peripheral TFCC lesion, and type 2 C the most frequent central lesion, supporting previous observations [23–25]. Some lesions did not fit Palmer’s classification, confirming the need for a broader classification, as suggested by Herzberg et al. [19].

Additional findings, including SLL, LTL injuries, and cartilage damage, were frequently found across many patients. While these findings argue for total wrist arthroscopy, they also necessitate caution. As previously emphasized, the indication for diagnostic arthroscopy must be carefully considered, as unexpected lesions of unclear clinical relevance can be found, increasing the risk of overdiagnosis and overtreatment if not correlated with the clinical picture [26–28].

During arthroscopy, 26.4% of patients showed no TFCC lesion, and 16.0% had not any arthroscopic abnormalities, reinforcing the diagnostic complexity of ulnar-sided wrist pain and supporting non-surgical management when appropriate.

We observed that 57.1% of patients with a confirmed TFCC lesion ultimately underwent either open TFCC repair or ulnar shortening osteotomy, depending on the specific anatomical structure involved—namely, the radioulnar ligaments or the central disc, respectively. As anticipated, TFCC repair was most frequently performed in patients with Palmer type 1B lesions, while the rate of repair was lower among other Palmer type 1 subtypes. Surgical decision-making was primarily influenced by the type of lesion and the stability of the distal radioulnar joint [29]. Ulnar shortening osteotomy was most commonly indicated in patients with Palmer type 2 lesions, typically based on clinical and radiographic evidence of ulnar impaction syndrome. A higher Palmer 2 classification grade correlated with an increased likelihood of surgical intervention, reflecting the progressive nature of damages found and associated symptom burden [2].

The complication rate (7.4%) was slightly higher than previous reports (4.7-6.0%) [10, 11], partly due to broad inclusion of minor issues. Most complications were mild; however, rare but serious events such as pneumothorax following a brachial plexus block were noted, underscoring that wrist arthroscopy carries non-negligible risks.

The strengths of this study include the large cohort and comprehensive reporting of patients suspected of TFCC lesions. However limitations involve the retrospective design, heterogeneity in surgical practice, subjective interpretation of arthroscopy findings [12, 13], and no midcarpal assessment in 274 patients (54.8%).

In conclusion, wrist arthroscopy is decisive in diagnosing or excluding TFCC lesions and assessing the type and severity of TFCC lesion in addition to detecting concomitant lesions. Furthermore, it is important to know that a negative finding helps to decide on treatment strategy. Therefore, both positive and negative findings play a pivotal role in the management of ulnar-sided wrist pain.

## Data Availability

No datasets were generated or analysed during the current study.
