# Correction: Diagnostic wrist arthroscopy: findings in patients suspected of TFCC lesions

**DOI:** 10.1007/s00402-025-06069-3

**Published:** 2025-09-29

**Authors:** Lyse van Wijk, Sandesh Kasie, Claire Koeyvoets, Sebastiaan Souer, Hand-Wrist Study Group, Steven Hovius, Brigitte van der Heijden

**Affiliations:** 1 Department of Plastic, Reconstructive and Hand Surgery, Radboud University Nijmegen Medical Centre, Nijmegen, Netherlands; 2 Hand and Wrist Center, Xpert Clinics, Amsterdam, Netherlands; 3 Department of Plastic, Reconstructive and Hand Surgery, Jeroen Bosch Ziekenhuis, ‘s-Hertogenbosch, Netherlands

**Correction: Archives of Orthopaedic and Trauma Surgery (2025) 145:399**

10.1007/s00402-025-06002-8

In the original version of this article, the order in which the authors appeared in the author list was incorrectly given as

Lyse van Wijk · Sandesh Kasie · Claire Koeyvoets · Sebastiaan Souer · Steven Hovius · Brigitte van der Heijden · Hand-Wrist Study Group

where it should have been

 Lyse van Wijk · Sandesh Kasie · Claire Koeyvoets · Sebastiaan Souer · Hand-Wrist Study Group · Steven Hovius · Brigitte van der Heijden

The original article has been corrected.

