# Correction: Diagnostic wrist arthroscopy: findings in patients suspected of TFCC lesions

**DOI:** 10.1007/s00402-025-06117-y

**Published:** 2025-11-21

**Authors:** Lyse van Wijk, Sandesh Kasie, Claire Koeyvoets, Sebastiaan Souer, Hand-Wrist Study Group, Steven Hovius, Brigitte van der Heijden

**Affiliations:** 1 Department of Plastic, Reconstructive and Hand Surgery, Radboud University Nijmegen Medical Centre, Nijmegen, Netherlands; 2 Hand and Wrist Center, Xpert Clinics, Amsterdam, Netherlands; 3 Department of Plastic, Reconstructive and Hand Surgery, Jeroen Bosch Ziekenhuis, ‘s-Hertogenbosch, Netherlands

**Correction: Archives of Orthopaedic and Trauma Surgery (2025) 145:399**

10.1007/s00402-025-06002-8

In the original version of this article, under methods section the lines which previously appeared as

“We conducted a retrospective cohort study of patients undergoing diagnostic wrist arthroscopy between 2012 and 2021 at <blinded> in <blinded>. <Blinded> comprises 28 clinics providing specialized hand and wrist care”.

but now it should be appeared as

“We conducted a retrospective cohort study of patients undergoing diagnostic wrist arthroscopy between 2012 and 2021 at Xpert Clinics in The Netherlands. Xpert Clinics comprises 28 clinics providing specialized hand and wrist care”.

